# Poster Session II - A243 PERINATAL NUTRIENT CONSUMPTION DIFFERS IN PREGNANT WOMEN WITH AND WITHOUT INFLAMMATORY BOWEL DISEASES

**DOI:** 10.1093/jcag/gwaf042.243

**Published:** 2026-02-13

**Authors:** K Xiao, R Dang, R Wu, K O’Connor, A Epstein-Shapiro, F Maiocco, V Srikanth, M Sobel, C Maxwell, V W Huang

**Affiliations:** Medicine, University of Toronto Temerty Faculty of Medicine, Toronto, ON, Canada; McMaster University, Hamilton, ON, Canada; Division of Gastroenterology and Hepatology, University of Toronto, Toronto, ON, Canada; Division of Gastroenterology, Department of Medicine, Sinai Health, Toronto, ON, Canada; Department of Nursing, University of Toronto, Toronto, ON, Canada; Division of Gastroenterology, Department of Medicine, Sinai Health, Toronto, ON, Canada; Division of Gastroenterology, Department of Medicine, Sinai Health, Toronto, ON, Canada; Division of Gastroenterology, Department of Medicine, Sinai Health, Toronto, ON, Canada; Division of Gastroenterology, Department of Medicine, Sinai Health, Toronto, ON, Canada; Division of Gastroenterology, Department of Medicine, Sinai Health, Toronto, ON, Canada

## Abstract

**Background:**

Inflammatory bowel disease (IBD) is characterized by chronic inflammation of the gastrointestinal tract with Crohn’s disease (CD) and ulcerative colitis (UC) as the two main phenotypes. IBD is often diagnosed during the reproductive years and presents to be a challenge due to the limited evidence of its impact during the peripartum period. Macronutrient intake during pregnancy is critical in women with IBD because chronic intestinal inflammation and malabsorption can impair maternal nutrient status and fetal growth.

**Aims:**

This study aims to compare the dietary patterns of women with and without IBD and their infant growth until 6 months (6M) postpartum.

**Methods:**

In total, 34 pregnant women with IBD and 12 pregnant women without IBD were recruited into our single-center prospective study at Mount Sinai Hospital, a tertiary care centre in Toronto with a high-risk pregnancy and IBD clinic. Participants completed a 3-day food intake diary at 3 months (3M) postpartum. Maternal nutrient intake and food group classification were assessed using ESHA Food Processor Nutrition Genesis. Infant growth measurements were compared to the World Health Organization’s standardized growth curve.

**Results:**

Twenty-four women with IBD and 9 women without IBD completed the 3M dietary assessment. Women with IBD had lower consumption of protein compared to women without IBD (80.8 ± 21.4 g vs 89.0 ± 38.2 g, p = 0.414), less carbohydrate (271.4 ± 63.7 g vs 307.9 ± 111.3 g, p = 0.79), less fiber (19.7 ± 8.2 g vs 21.3 ± 7.1 g, p = 0.32), but more fats (95.7 ± 44.2 g vs 90.6 ± 29.4 g, p = 0.79) daily at 3M. Women with IBD’s diet consisted of 10% fewer vegetables at 1M (p = 0.10) and 3M (p = 0.15) postpartum. Infants born from women with IBD who met their recommended fat intake had lower weight-for-age (WFA) Z-scores compared to infants of women without IBD who met their fat intake at 3M (p = 0.48) and 6M (p = 0.47). There was no statistical difference in the WFA and length-for-age (LFA) Z-scores up to 6M.

**Conclusions:**

Women with IBD consumed more fats, but less protein, carbohydrates, and fiber than women without IBD. Infants born to women with IBD had a lower WFA and similar LFA Z-scores compared to infants of women without IBD. Future research will investigate the role of nutritional interventions and dietary counseling in optimizing pregnancy and infant outcomes in women with IBD.

**Funding Agencies:**

American College of Gastroenterology Resident Grant, University of Toronto’s Division of Gastroenterology Resident Grant, University of Toronto’s Department of Medicine Resident Grant, CAN-TAP-TALENT

